# Supplementary material for: Specialized Pediatric Palliative Care Services in Pediatric Hematopoietic Stem Cell Transplant Centers
Source: Children (Basel). 2021 Jul 21;8(8):615. doi: 10.3390/children8080615 (PMC8393700; doi:10.3390/children8080615)
Supplement: Supplementary file 1 [file children-08-00615-s001.zip › children-1277095-supplementary.pdf]

Table S1 Health Care Professionals Responses on the survey

Questions	Responses	All respondents (*n* = 98)	Physician (*n* = 42)	Nurse (*n* = 40)	Affiliated (*n* = 16)
What is your country?	Belarus	1 (1)	1 (2.4)	0 (0)	0 (0)
	Belgium	7 (7.1)	2 (4.8)	3 (7.5)	2 (12.5)
	Brazil	1 (1)	1 (2.4)	0 (0)	0 (0)
	Czech Republic	1 (1)	1 (2.4)	0 (0)	0 (0)
	Denmark	4 (4.1)	1 (2.4)	2 (5)	1 (6.2)
	France	8 (8.2)	3 (7.1)	4 (10)	1 (6.2)
	Germany	8 (8.2)	4 (9.5)	4 (10)	0 (0)
	Hungary	3 (3.1)	1 (2.4)	1 (2.5)	1 (6.2)
	Ireland	1 (1)	1 (2.4)	0 (0)	0 (0)
	Israel	5 (5.1)	1 (2.4)	3 (7.5)	1 (6.2)
	Italy	5 (5.1)	2 (4.8)	3 (7.5)	0 (0)
	Lithuania	2 (2)	1 (2.4)	1 (2.5)	0 (0)
	Netherlands	4 (4.1)	2 (4.8)	1 (2.5)	1 (6.2)
	Norway	2 (2)	0 (0)	1 (2.5)	1 (6.2)
	Pakistan	1 (1)	1 (2.4)	0 (0)	0 (0)
	Poland	3 (3.1)	1 (2.4)	1 (2.5)	1 (6.2)
	Russia	3 (3.1)	1 (2.4)	1 (2.5)	1 (6.2)
	Saudi Arabia	1 (1)	1 (2.4)	0 (0)	0 (0)
	Spain	18 (18.4)	8 (19)	7 (17.5)	3 (18.8)
	Sweden	6 (6.1)	2 (4.8)	3 (7.5)	1 (6.2)
	Switzerland	2 (2)	1 (2.4)	1 (2.5)	0 (0)
	Turkey	1 (1)	0 (0)	0 (0)	1 (6.2)
	UK	11 (11.2)	6 (14.3)	4 (10)	1 (6.2)
Pediatric palliative services according to WHO should provide symptom, emotional, social and spiritual support from the time of diagnosis of a life threatening or life shortening illness.Do you agree that HSCT patients in light of the risk of Transplant Related Mortality (TRM) and relapse should have access to SPPCS?	No	8 (8.2)	3 (7.1)	3 (7.5)	2 (12.5)
	Yes	90 (91.8)	39 (92.9)	37 (92.5)	14 (87.5)
What time point do you consider initial referral to SPPCS appropriate?	At the onset of terminal care	23 (23.5)	12 (28.6)	7 (17.5)	4 (25)
	At the onset of a life threatening event(s)	13 (13.3)	7 (16.7)	3 (7.5)	3 (18.8)
	At the time of admission to HSCT unit	13 (13.3)	3 (7.1)	8 (20)	2 (12.5)
	At the time of admission to ICU	4 (4.1)	0 (0)	4 (10)	0 (0)
	At the time of difficult symptom control	21 (21.4)	11 (26.2)	5 (12.5)	5 (31.2)
	At the time of original diagnosis	20 (20.4)	7 (16.7)	11 (27.5)	2 (12.5)

	Not needed (can be provide by the unit without recourse to SPPCS)	3 (3.1)	1 (2.4)	2 (5)	0 (0)
	To provide bereavement counseling	1 (1)	1 (2.4)	0 (0)	0 (0)
**Please rank how important you consider the following services provided by SPPCS by ticking the appropriate answer**					
Pain management -	extremely important (+2)	80 (81.6)	32 (76.2)	33 (82.5)	15 (93.8)
	very important (+1)	16 (16.3)	9 (21.4)	6 (15)	1 (6.2)
	neutral (0)	1 (1)	1 (2.4)	0 (0)	0 (0)
	not very important (-1)	0 (0)	0 (0)	0 (0)	0 (0)
	not necessary (-2)	1 (1)	0 (0)	1 (2.5)	0 (0)
Other symptom control -	extremely important (+2)	37 (37.8)	15 (35.7)	19 (47.5)	3 (18.8)
	very important (+1)	51 (52)	24 (57.1)	19 (47.5)	8 (50)
	neutral (0)	9 (9.2)	3 (7.1)	1 (2.5)	5 (31.2)
	not very important (-1)	0 (0)	0 (0)	0 (0)	0 (0)
	not necessary (-2)	1 (1)	0 (0)	1 (2.5)	0 (0)
Emotional support -	extremely important (+2)	50 (51)	17 (40.5)	23 (57.5)	10 (62.5)
	very important (+1)	41 (41.8)	22 (52.4)	13 (32.5)	6 (37.5)
	neutral (0)	6 (6.1)	3 (7.1)	3 (7.5)	0 (0)
	not very important (-1)	0 (0)	0 (0)	0 (0)	0 (0)
	not necessary (-2)	1 (1)	0 (0)	1 (2.5)	0 (0)
Spiritual support -	extremely important (+2)	26 (26.5)	12 (28.6)	10 (25)	4 (25)
	very important (+1)	42 (42.9)	13 (31)	20 (50)	9 (56.2)
	neutral (0)	27 (27.6)	16 (38.1)	8 (20)	3 (18.8)
	not very important (-1)	2 (2)	1 (2.4)	1 (2.5)	0 (0)
	not necessary (-2)	1 (1)	0 (0)	1 (2.5)	0 (0)
Ethical guidance -	extremely important (+2)	35 (35.7)	14 (33.3)	15 (37.5)	6 (37.5)
	very important (+1)	36 (36.7)	13 (31)	16 (40)	7 (43.8)
	neutral (0)	22 (22.4)	13 (31)	7 (17.5)	2 (12.5)
	not very important (-1)	3 (3.1)	2 (4.8)	0 (0)	1 (6.2)
	not necessary (-2)	2 (2)	0 (0)	2 (5)	0 (0)
Ensuring quality of life -	extremely important (+2)	71 (72.4)	28 (66.7)	30 (75)	13 (81.2)
	very important (+1)	24 (24.5)	12 (28.6)	9 (22.5)	3 (18.8)
	neutral (0)	2 (2)	2 (4.8)	0 (0)	0 (0)
	not very important (-1)	0 (0)	0 (0)	0 (0)	0 (0)
	not necessary (-2)	1 (1)	0 (0)	1 (2.5)	0 (0)
Facilitating shared decision making with the child -	extremely important (+2)	52 (53.1)	18 (42.9)	24 (60)	10 (62.5)
	very important (+1)	35 (35.7)	17 (40.5)	14 (35)	4 (25)
	neutral (0)	10 (10.2)	7 (16.7)	1 (2.5)	2 (12.5)
	not very important (-1)	1 (1)	0 (0)	1 (2.5)	0 (0)
	not necessary (-2)	0 (0)	0 (0)	0 (0)	0 (0)
Facilitating shared decision making with parent(s) -	extremely important (+2)	63 (64.3)	26 (61.9)	25 (62.5)	12 (75)
	very important (+1)	30 (30.6)	14 (33.3)	13 (32.5)	3 (18.8)
	neutral (0)	4 (4.1)	2 (4.8)	1 (2.5)	1 (6.2)
	not very important (-1)	1 (1)	0 (0)	1 (2.5)	0 (0)

	not necessary (-2)	0 (0)	0 (0)	0 (0)	0 (0)
Facilitating communication with parents about end of life issues -	extremely important (+2)	70 (71.4)	25 (59.5)	30 (75)	15 (93.8)
	very important (+1)	21 (21.4)	13 (31)	7 (17.5)	1 (6.2)
	neutral (0)	6 (6.1)	4 (9.5)	2 (5)	0 (0)
	not very important (-1)	1 (1)	0 (0)	1 (2.5)	0 (0)
	not necessary (-2)	0 (0)	0 (0)	0 (0)	0 (0)
Facilitating communication with the child about end of life issues -	extremely important (+2)	53 (54.1)	16 (38.1)	25 (62.5)	12 (75)
	very important (+1)	32 (32.7)	18 (42.9)	11 (27.5)	3 (18.8)
	neutral (0)	11 (11.2)	7 (16.7)	3 (7.5)	1 (6.2)
	not very important (-1)	2 (2)	1 (2.4)	1 (2.5)	0 (0)
	not necessary (-2)	0 (0)	0 (0)	0 (0)	0 (0)
Supporting the HSCT team in difficult decision making process around end life issues -	extremely important (+2)	49 (50)	18 (42.9)	22 (55)	9 (56.2)
	very important (+1)	37 (37.8)	18 (42.9)	14 (35)	5 (31.2)
	neutral (0)	9 (9.2)	5 (11.9)	3 (7.5)	1 (6.2)
	not very important (-1)	1 (1)	0 (0)	1 (2.5)	0 (0)
	not necessary (-2)	2 (2)	1 (2.4)	0 (0)	1 (6.2)
Bereavement counseling -	extremely important (+2)	38 (38.8)	13 (31)	17 (42.5)	8 (50)
	very important (+1)	46 (46.9)	21 (50)	17 (42.5)	8 (50)
	neutral (0)	11 (11.2)	7 (16.7)	4 (10)	0 (0)
	not very important (-1)	2 (2)	0 (0)	2 (5)	0 (0)
	not necessary (-2)	1 (1)	1 (2.4)	0 (0)	0 (0)
Facilitating interdisciplinary care -	extremely important (+2)	47 (48)	18 (42.9)	21 (52.5)	8 (50)
	very important (+1)	37 (37.8)	16 (38.1)	14 (35)	7 (43.8)
	neutral (0)	10 (10.2)	7 (16.7)	3 (7.5)	0 (0)
	not very important (-1)	4 (4.1)	1 (2.4)	2 (5)	1 (6.2)
	not necessary (-2)	0 (0)	0 (0)	0 (0)	0 (0)
Advance care plan provision -	extremely important (+2)	36 (36.7)	11 (26.2)	18 (45)	7 (43.8)
	very important (+1)	43 (43.9)	21 (50)	16 (40)	6 (37.5)
	neutral (0)	15 (15.3)	8 (19)	5 (12.5)	2 (12.5)
	not very important (-1)	2 (2)	0 (0)	1 (2.5)	1 (6.2)
	not necessary (-2)	2 (2)	2 (4.8)	0 (0)	0 (0)
Sum of the scores	median [IQR]	20 [15-24]	17 [13.2-22.8]	22.5 [15.8-25]	20.5 [19-23.5]
	(range)	(-21-28)	(3-28)	(-21-28)	(10-28)
Do you consider research in pediatric palliative care necessary?	No	3 (3.1)	2 (4.8)	1 (2.5)	0 (0)
	Yes	95 (96.9)	40 (95.2)	39 (97.5)	16 (100)
Do you consider research in pediatric palliative care important?	No	2 (2)	1 (2.4)	1 (2.5)	0 (0)
	Yes	96 (98)	41 (97.6)	39 (97.5)	16 (100)
Do you consider research in pediatric palliative care ethical?	No	1 (1)	1 (2.4)	0 (0)	0 (0)
	Yes	97 (99)	41 (97.6)	40 (100)	16 (100)
Answers are described as number (percentage- by column, conditionally to the profession) or median [IQR]					

Table S2 Physicians Responses on the survey

Questions	Responses	Physician (*n* = 42)
JACIE accredited?	No	15 (36.6)

	Yes	26 (63.4)
	missing	1
Does your HSCT unit have access to specialist pediatric palliative care services (SPPCS)?	No	7 (17.1)
	Yes	34 (82.9)
	missing	1
Does your HSCT unit routinely refer HSCT children to specialist pediatric palliative care services (SPPCS)?	No (including the No at Q4)	23 (56.1)
	Yes	18 (43.9)
	missing	1
**If Yes: Please indicate the type and number of staff involved in SPPCS team**		
Physician dedicated in palliative care	No	1 (6.2)
	Yes	15 (93.8)
	missing	2 on 18
Nurse dedicated to palliative care	No	2 (13.3)
	Yes	13 (86.7)
	missing	3 on 18
Social worker	No	3 (18.8)
	Yes	13 (81.2)
	missing	2 on 18
Play therapist	No	2 (13.3)
	Yes	13 (86.7)
	missing	3 on 18
Educationalist	No	5 (35.7)
	Yes	9 (64.3)
	missing	4 on 18
Pastor or other spiritual specialist	No	6 (40)
	Yes	9 (60)
	missing	3 on 18
Ethicist	No	10 (76.9)
	Yes	3 (23.1)
	missing	5 on 18
Pain specialist	No	1 (6.2)
	Yes	15 (93.8)
	missing	2 on 18
Other (please print)	care coordinator	1 (16.7)
	on an as needed basis	1 (16.7)
	Play therapist is a psychologist. The pastor is not in the team but he is available when needed	1 (16.7)
	psycholog	1 (16.7)
	psychologist	1 (16.7)
	Psycologist	1 (16.7)
**If no: Who provides pediatric palliative care?**		
Individual transplant physician in charge	No	1 (5.3)
	Yes	18 (94.7)
	missing	4 on 23
Adult palliative services	No	14 (77.8)
	Yes	4 (22.2)

	missing	5 on 23
Nurse led team	No	11 (61.1)
	Yes	7 (38.9)
	missing	5 on 23
Other (please state)	No	8 (50)
	Yes	8 (50)
	missing	7 on 23
Detail	Acute pain team and paediatric hospice team	1 (14.3)
	Care Team	1 (14.3)
	dedicated palliative referent physician and nurse for pediatrics	1 (14.3)
	Pain team (anesthesist)	1 (14.3)
	pediatric palliative physician	1 (14.3)
	Physicians of paediatric palliative service	1 (14.3)
	Psychologist	1 (14.3)
	missing	1 on 8
**Does your HSCT unit have access to?**		
Specialized pediatric palliative home care treatment facilities	No	4 (20)
	Yes	16 (80)
	missing	22
Pediatric hospice	No	17 (41.5)
	Yes	24 (58.5)
	missing	1
Specialized bereavement services	No	18 (43.9)
	Yes	23 (56.1)
	missing	1
Out of hours palliative care team	No	11 (28.9)
	Yes	27 (71.1)
	missing	4
Are Institutional guidelines available for pediatric palliative care?	No	14 (37.8)
	Yes	23 (62.2)
	missing	5
Are National guidelines available for pediatric palliative care ?	No	20 (58.8)
	Yes	14 (41.2)
	missing	8
Do you routinely use Advanced Care Planning (a process that enables individuals to make plans about their future health care) in your HSCT unit?	No	24 (60)
	Yes	16 (40)
	missing	2
